# Assistance to Victims of Sexual Violence in a Referral Service: A 10-Year Experience

**DOI:** 10.1055/s-0041-1740474

**Published:** 2022-01-29

**Authors:** Gabriel Ramalho de Jesus, Natália Pavoni Rodrigues, Giordana Campos Braga, Renata Abduch, Patricia Pereira dos Santos Melli, Geraldo Duarte, Silvana Maria Quintana

**Affiliations:** 1 Gynecology And Obstetrics Department, Faculdade de Medicina de Ribeirão Preto, Universidade de São Paulo, Ribeirão Preto, SP, Brazil

**Keywords:** sex offenses, rape, sexually transmitted diseases, violence against women, pregnancy unwanted, delitos sexuais, estupro, doenças sexualmente transmissíveis, violência contra a mulher, gravidez não desejada

## Abstract

**Objective** To evaluate the assistance provided to women victims of sexual violence and their participation in the follow-up treatment after the traumatic event, presenting a sociodemographic profile, gynecological background, and circumstances of the event, and reporting the results, acceptance, and side effects of prophylaxis for sexually transmitted infections (STIs) and pregnancy.

**Methods** A retrospective cohort study comprising the period between 2007 and 2016. All women receiving medical care and clinical follow-up after a severe episode of sexual violence were included. Records of domestic violence, male victims, children, and adolescents who reported consensual sexual activity were excluded. The present study included descriptive statistics as frequencies and percentages.

**Results** A total of 867 medical records were reviewed and 444 cases of sexual violence were included. The age of the victims ranged from 10 to 77 years old, most of them self-declared white, with between 4 and 8 years of education, and denying having a sexual partner. Sexual violence occurred predominantly at night, on public thoroughfare, being committed by an unknown offender. Most victims were assisted at the referral service center within 72 hours after the violence, enabling the recommended prophylaxis. There was high acceptance of antiretroviral therapy (ART), although half of the users reported side effects. Seroconversion to human immunodeficiency virus (HIV) or to hepatitis B virus (HBV) was not detected in women undergoing prophylaxis.

**Conclusion** In the present cohort, the profile of victims of sexual violence was low-educated, young, white women. The traumatic event occurred predominantly at night, on public thoroughfare, being committed by an unknown offender. Assistance within the first 72 hours after sexual violence enables the healthcare center to provide prophylactic interventions against STIs and unwanted pregnancies.

## Resumo

**Objetivo** Avaliar a assistência prestada às mulheres vítimas de violência sexual e seu acompanhamento após o evento traumático, caracterizando o perfil sociodemográfico, antecedentes ginecológicos e circunstâncias do evento, além de relatar a aceitação e os efeitos colaterais da profilaxia para infecções sexualmente transmissíveis (ISTs) e a ocorrência de gravidez resultante da violência sexual.

**Métodos** Estudo de coorte retrospectivo compreendendo o período entre 2007 e 2016. Foram incluídas todas as mulheres em acompanhamento médico e clínico após episódio de violência sexual. Foram excluídos registros de violência doméstica, vítimas do sexo masculino e crianças e adolescentes que relataram atividade sexual consensual. O estudo incluiu estatísticas descritivas, com frequências e percentuais.

**Resultados** Foram revisados 867 prontuários e 444 casos de violência sexual foram incluídos. A faixa etária foi 10 a 77 anos; a maioria das vítimas se autodeclarou branca, com entre 4 e 8 anos de escolaridade, e negou ter um parceiro sexual fixo. A violência sexual ocorreu predominantemente à noite, em via pública, por um agressor desconhecido. A maioria foi atendida no serviço de referência em até 72 horas após a violência, possibilitando profilaxias preconizadas. Houve alta aceitação da terapia antirretroviral (TARV), embora metade das usuárias relatasse efeitos colaterais. A soroconversão para o vírus da imunodeficiência humana (HIV, na sigla em inglês) ou para o vírus da hepatite B (HBV, na sigla em inglês) não foi detectada entre as vítimas.

**Conclusão** Nesta coorte, o perfil das vítimas de violência sexual foi de mulheres brancas, de baixa escolaridade, e jovens. O evento traumático ocorreu predominantemente à noite, em via pública, por um agressor desconhecido. A assistência nas primeiras 72 horas após a violência sexual permite que o serviço de saúde realize intervenções profiláticas contra ISTs e gravidez indesejada.

## Introduction

According to the World Health Organization (WHO), 1 sexual violence is a serious public health problem, being defined as any sexual act, attempt to obtain a sexual act, unwanted sexual comments or advances, using mental or physical coercion or aggression, in any setting, including but not limited to the household and work environments. In addition to the immediate risks resulting from sexual violence, such as sexually transmitted infections (STIs) 2 3 and unwanted pregnancies, a high percentage of victims develop mental health disorders in the medium- and long-term, with a strong tendency to present psychiatric disorders, social isolation, use of psychoactive substances, and suicide. 4 5

National studies showed that 20% of the population have already experienced sexual violence. 6 This type of violence is twice as frequent in the female population, being estimated that up to 40% of women had a violent sexual experience. 6 7 8 9 In female adolescents, the prevalence of this type of violence is six times higher when compared with adult women. 10

The elaboration of technical norms and clinical protocols for the reception, care, and notification of violence is based on the international guidelines of the WHO. 11 12 In Brazil, beginning in the 1980s, the Ministry of Health standardized assistance for people who suffered sexual violence. These guidelines were updated over the years, and the last ones were published in 2014 13 and 2015, 14 having been elaborated in partnership with the Health Departments of the federation units, as well as with scientific societies and social movements.

Health system organization and professional training are essential to improve the reception and healthcare of victims of violence, consequently decreasing its damages. The assistance in the health service must be immediate and, if possible, performed by a multidisciplinary team with the participation of physicians, nurses, social workers, and psychologists. 11 12 13 14 Thus, it is possible to welcome, assist, conduct clinical and laboratory tests, and administer emergency contraception and chemoprophylaxis for human immunodeficiency virus (HIV) and other STIs. Immediate prophylaxis is an effective measure that must be available in every health center assisting these victims. It is also possible to offer psychosocial support, being aware of the importance of training the professionals who work directly with these victims, improving their skills and technical capacity for the treatment of victims of sexual offense. However, there are few services in Brazil with a specialized profile, most of them linked to referral or university hospitals. 15

Within this context, the Hospital das Clínicas da Faculdade de Medicina de Ribeirão Preto da Universidade de São Paulo (HCFMRP-USP, in the Portuguese acronym), Ribeirão Preto, state of São Paulo, Brazil, offers the Serviço de Atenção às Vítimas de Violência Doméstica e Agressão Sexual (Assistance Service for Victims of Domestic Violence and Sexual Assault [SEAVIDAS, in the Portuguese acronym]) to the population of the Regional Health Directorate (Direção Regional de Saúde - DRS) XIII. This regional health service comprises 26 cities with an estimated population of 1,450,000 people, with 700,000 inhabitants in Ribeirão Preto, state of São Paulo, Brazil. This service is maintained with funding from the Government of the State of São Paulo, aiming at providing comprehensive health care to victims of domestic violence and sexual assault at the tertiary level, referred by primary health care centers, by social assistance units, by legal units or spontaneous search for care. In addition, the implemented structure is interdisciplinary and multiprofessional, with physicians, nurses, psychologists, and social workers providing appropriate care for the victims.

The patient flow recommended by the SEAVIDAS expects situations of violence classified as severe or chronic. Severe violence is characterized as occurring in the previous 72 hours (up to a maximum of 5 days) and requires immediate prophylaxis conducted in a hospital environment, in an emergency regime, not requiring the referral regulation system. After the initial reception, the victim joins the assistance service to continue a follow-up treatment for 6 months. Chronic violence is characterized as having occurred > 72 hours before (or > 5 days) and/or being recurrent. In these cases, the patients are received at the center referred via state regulation and receive assistance at an outpatient service unit. The SEAVIDAS also carries out the ending of the pregnancy resulting from sexual violence (according to Law 12,015/2009). 16

The objectives of the present study are to characterize the sociodemographic profile of women victims of severe sexual violence treated at the SEAVIDAS-HCFMRPUSP, to know, from the first medical visit, the circumstances in which the violent event occurred, and to evaluate the effectiveness of the prophylaxis protocols for STIs and pregnancy resulting from rape, and the participation of the victims in the follow-up treatment after the violence.

## Methods

This is a retrospective cohort study conducted between 2007 and 2016 that included all assistance provided to women at the SEAVIDAS-HCFMRP USP after an immediate act of sexual violence. Cases of domestic violence, physical or verbal violence without sexual offense, male victims, premenarchal girls, and girls < 14 years of old who reported consensual sexual intercourse were excluded.

By reviewing the medical records, some variables in the assistance of severe episodes were evaluated, such as sociodemographic (age, origin, color/race, marital status, paid activity, education); medical (pregnancies, sexual activity, use of contraception, psychiatric conditions); circumstances of the abuse (place, time, number of offenders, relationship with the offender), and legal aspects (medical examination and police report). In the initial medical care, the time of arrival at the service, concurrence of trauma or physical aggression, previous diseases, and postexposure prophylaxis (contraception and STIs) were checked.

The SEAVIDAS assistance protocol for women victims of sexual violence is based on the Technical Guidelines of the Brazilian Ministry of Health. 13 It is a multidisciplinary service, with compulsory notification to health and police authorities. When the victim is < 18 years old, a police report and a medical-legal report made by a physician from the Medical Examiner's Office (Instituto Médico Legal [IML, in the Portuguese acronym]) with evidence collection of vaginal content for future identification of the DNA of the offender are mandatory.

After anamnesis and physical examination, the patient undergoes a pregnancy test, hepatitis B surface antigen (HBsAg), hepatitis C, HIV, and syphilis serology, and biochemistry tests (liver enzymes, kidney function, blood glucose, and blood count). In the assistance service, the victim receives prophylaxis consisting of levonorgestrel or a copper intrauterine device (IUD) for emergency contraception; azithromycin and ceftriaxone for bacterial infections; hepatitis B virus (HBV) vaccine and immunoglobulin (according to vaccination status); HIV antiretroviral therapy (ART) provided by the Brazilian Ministry of Health; tetanus vaccine; and metronidazole for trichomoniasis.

The patients are reassessed in an outpatient setting 7 to 10 days after the traumatic event to analyze their emotional state, emergency room test results, and ART acceptance. Medical and psychological follow-up is also offered for 6 months after the violence, and STI serology is retested 3 and 6 months after the traumatic event. The described reception and interventions are fundamental to minimize the damage caused by sexual violence to the life of a woman.

The present study was approved by the Research Ethics Committee of the HCFMRP USP, under the number 2,283,582/2017.

## Results

**Fig. 1:**
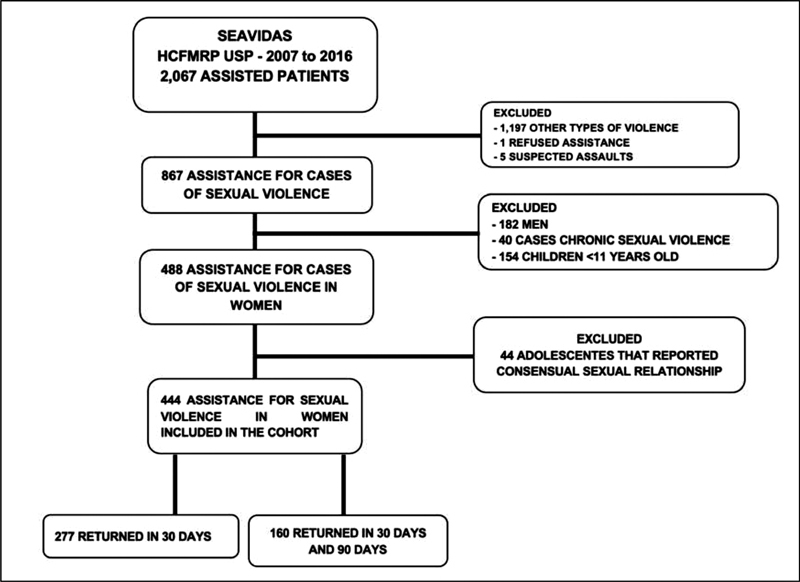
Assistance flowchart for cases of violence at SEAVIDAS-HCRP between 2007 and 2016.

Between 2007 and 2016, 2,067 victims of some type of violence were treated at the SEAVIDAS-HCFMRP USP. Of these, 867 were victims of sexual violence, but after applying the inclusion and exclusion criteria, 444 medical records were analyzed for sexual violence against women at the SEAVIDAS-HCRP. This process is described in the flowchart in Figure 1 .

Table 1 shows the profile of the 444 victims of sexual violence. The age group between 20 and 59 years old comprised the highest number of cases (49.55%), and the mean age of the victims was 24.1 years old (median of 20 years old). Most victims declared to be white, were from the city of Ribeirão Preto, state of São Paulo, Brazil, had between 4 and 8 years of education, did not have a paid activity, and did not have a steady sexual partner. Regarding their gynecological and obstetric histories, 155 victims (35.42%) denied having started sexual activity before the rape, 286 victims (65%) were nulliparous, and 325 (74.55%) did not use contraceptive methods. A diagnosis of psychiatric disorder prior to victimization (including use of psychoactive substances, cognitive impairment, and psychic comorbidities) was positive in 100 women (23.11%); however, it was not possible to access data on the mental health follow-up of these patients.

**Table 1:** Distribution of 444 women victims of sexual violence according to the sample characterization

Data	*n*	%
Age (years old)		
11–14	97	21.85%
15–19	117	26.35%
20–59	220	49.55%
≥ 60	10	2.25%
Color		
White	315	71.11%
Not white	128	28.89%
Education		
Illiterate	26	6.47%
4 to 8 years	205	51.00%
9 to 12 years	132	32.84%
Higher/technical education	39	9.70%
Paid activity		
Yes	134	31.16%
No	296	68.84%
Origin		
Ribeirão Preto	239	53.83%
DRS XIII cities	192	43.24%
Other cities	13	2.93%
Sexual partner		
Yes	53	12.18%
No	382	87.82%
Previous sexual activity		
Yes	155	35.42%
No	279	64.58%
Parity		
Nulliparous	286	65.00%
Primiparous	43	9.77%
Multiparous	111	25.23%
Use of contraception		
Yes	111	25.46%
No	325	74.54%
Psychiatric disorder		
Yes	100	23.11%
No	336	76.89%

Table 2 shows the circumstances of the sexual assault. Most of them happened at night, outdoors, with a single unknown offender. There was a record of use of psychoactive drugs, voluntary or forced by the offender, in 33 patients (7.69%), and of use of condoms by the offender in 14 victims (3.21%).

**Table 2:** Distribution of 444 women victims of sexual violence according to the circumstances of the traumatic event

Data	*n*	Frequency
Time		
Day	126	30.9%
Night	197	66.9%
False imprisonment	9	2.2%
Place		
Outdoors	167	38.3%
Victim's house	91	20.9%
Offender's house	69	15.8%
Rural area	27	6.2%
Other	82	18.8%
Number of offenders		
1	267	82.4%
2	33	10.2%
≥ 3	24	7.4%
Relationship with the offender		
Unknown	264	59.4%
Known	180	40.6%
Current partner	12	6.7%
Former partner	21	11.7%
Other	147	81.6%
Use of psychoactive drugs		
Yes	33	7.69%
No	396	92.31%
Use of contraceptive in the act		
Yes	14	3.21%
No	422	96.79%

The time of arrival at the health center to receive assistance after sexual violence was up to 72 hours for 346 victims (77.9%), with 257 (57.9%) having been assisted within the first 24 hours after the violence. In the initial care, the medical team observed a concomitance of physical aggression in 170 women (38.2%), and the evidence of recent hymen rupture in 75 (16.9%) of these was confirmed by an expert physician. A police report and medical-legal evaluation were carried out for 391 (88.1%) victims of sexual violence treated at the SEAVIDAS-HCRP.

Regarding prophylaxis at the first visit, 14 victims (3.1%) had a previous diagnosis of STI or had a rapid reagent test. Of these, four women had HIV, four were chronic carriers of the HBV, and six patients had reagent serology for syphilis. Therefore, these women did not receive prophylaxis for these infections. Emergency contraception was administered to 296 (66.7%) victims, ceftriaxone, azithromycin, and hepatitis B vaccine to 386 (86.9%), and ART to 331 (74.5%), as shown in Table 3 . Prophylaxis against trichomoniasis was prescribed in the outpatient visit 7 to 10 days after the traumatic event.

**Table 3:** Distribution of 444 women victims of sexual violence according to the received prophylaxis

Prophylaxis	*n*	%
Contraception	296	66.7%
STI		
ART	331	74.5%
Bacterial and HBV	386	86.9%

Abbreviations: ART, antiretroviral therapy; HBV, hepatitis B virus; STI, sexually transmitted infection.

Regarding ART acceptance, out of the 331 victims who accepted it, 253 used the therapy adequately (76.4%). However, 23.6% of the victims did not complete the 28 days of prophylaxis. Of the victims who used ART correctly, 49.5% reported gastrointestinal side effects such as nausea, vomiting, epigastric pain or lack of appetite, and 13.9% reported neurological symptoms such as headache, insomnia, drowsiness, or dizziness. Two patients presented hematological changes (anemia or leukopenia), and the antiretroviral medications were changed.

The outpatient follow-up after the traumatic event showed that 227 women (51.1%) returned in the 1 ^st^ month and 160 women (36%) returned in the 3 ^rd^ month. The prophylaxis result did not show seroconversion to HIV or HBV infection in the women who repeated serology, and all 6 patients with a syphilis serological diagnosis underwent a follow-up with venereal disease research laboratory (VDRL) retest according to the protocol recommended by the Brazilian Ministry of Health. In this sample, pregnancy was diagnosed in a victim of sexual violence who had not received emergency contraception because she arrived at the health service 5 days after the traumatic event.

## Discussion

A total of 444 cases of women victims of sexual violence within a 10-year period was studied. According to article 213 of the Brazilian Penal Code (1940), rape is the act of forcing someone, with violence or serious threat, to have carnal conjunction or to engage in or consent to a libidinous act. 16 17 The Brazilian Public Security Yearbook, published in 2020, 18 pointed out that there are 8 rapes per minute in Brazil. This number is certainly underestimated, as it corresponds to the number of cases reported to the police authorities. This underreporting is justified by the victim's fear of the offender, self-blame and shame, and the fear of being mistreated at police stations. 19 Although there are no consistent studies to confirm these numbers, estimates indicate that they can be up to 10 times higher, placing sexual violence as a public health problem worldwide.

The sociodemographic profile of the women treated at the SEAVIDAS-HCRP during the period studied was quite similar to the one described in the national and international literature, with a predominance of young women, self-declared white, without paid activity, and without a steady sexual partner. 5 15 Although violence against women, especially sexual, can occur at any age, it is much more frequent in adolescents and young adults. In the present case series, despite the wide age range of the victims (11 to 77 years old), the mean age was 24.4 years old, with a predominant age range between 11 and 19 years old, which corresponded to 43.9% of the victims, confirming the high number of adolescents who suffer sexual violence. This high number was described in other studies and can partly be explained by the vulnerability of this population, by the fact that the offender is part of the family circle of the victim, and by the difficulty adolescents have in assessing the risk of exposing themselves to situations that may culminate in sexual violence. 10

Although, under Brazilian law, sexual intercourse under the age of 14 is considered rape and classified as presumed violence (rape of a vulnerable individual) even with the consent of the victim, 16 the 44 girls < 14 years old who reported consensual sexual intercourse were not included in this analysis, since they were not considered victims of a traumatic event.

Regarding education, half of the victims reported having studied between 4 and 8 years and 32.8% had between 9 and 12 years of education. Although the population of the present cohort consists predominantly of young women, the low level of education reported by 51% of the victims is noteworthy. Most victims did not have any paid activity and did not have a sexual partner at the time of the assault. This profile can possibly be explained by the high number of adolescents in the present case series, since it is a period when they change partners frequently, and do not have a steady relationship or a paid activity.

Moreover, regarding the profiles of the victims, approximately a quarter of them reported having some kind of psychiatric disorder prior to the traumatic event, pointing out the vulnerability of this population. It is known that the prevalence of any type of violence is higher in the female population, since women are victims in 85% of cases of violence, and psychiatric disorders are more frequent in this population. 20 21 Therefore, it can be concluded that women with mental health disorders are at a greater risk of suffering sexual violence and should receive special attention from health and social service teams with a focus on preventive measures. It is once again reiterated that the impact of violence on psychological and behavioral aspects can aggravate pre-existing situations and persist throughout life with a negative impact on future sexual relationships. 21 22 23

In conclusion, the analyses of the origin of the victims of sexual violence treated at the SEAVIDAS-HCRP during the period studied showed the importance of the broad operation that this assistance service has in the region. Approximately half of the victims came from Ribeirão Preto, which is the largest city in this Regional Health Department, while 43% came from the other 25 cities in this Department. The structuring of a comprehensive health care system for people in situations of sexual violence is an important step to ensure healthcare promotion and prevention for this group. 13 14 For this flow to be established, the SEAVIDAS-HCRP conducted several activities to disseminate protocols and matrix support, in addition to training health professionals, playing a fundamental role in ensuring adequate reception and decreased damages for these victims.

The results of the present study indicate that most rapes occurred at night, in an external environment, and were committed by an unknown offender; these circumstances are similar to those described in other national studies, such as that by Labronici et al. 24 Although more than half of the cases of sexual violence (59.4%) were committed by an unknown offender, the high percentage of sexual violence committed by someone the victim knows (40.6%) is noteworthy. In the cases committed by a known offender, the current or former partner was responsible for 18.4% of the cases. These data are quite worrisome, as they point out that women suffer violence even in supposedly safe environments and raises the question of the invisibility of aggression within the marital relationship, as discussed by Schraiber et al. 25 and Dantas-Berger et al. 26 In these texts, the authors discuss intimate partner violence as a determinant of serious consequences and how proximity to the offender makes it difficult to report and seek the right care. These difficulties make it impossible for women to receive prophylaxis, losing the chance to prevent STIs and pregnancy.

The present study showed that a significant number of victims of sexual violence had also suffered physical violence at the first appointment. This data is highly relevant in the context of fighting violence against women, showing the need to support victims of violence. In addition, the forced or voluntary use of substances during the act occurred in 7.69% of the sample, demonstrating an additional vulnerability factor to which the patients may be exposed.

All victims were offered to file a police report (PR), which was mandatory for victims < 18 years old. In the present study, ∼ 90% of rape cases had a filed a PR, a result above the national average, pointing out the importance of the partnership between the SEAVIDAS-HCRP and the Secretariat for Public Security. It is important to highlight that, when filling in the PR, the Medical Examiner's Office is informed to conduct a forensic medical examination; that is, the victim will be examined by a physician and material will be collected for eventual identification of the DNA of the offender. However, it is difficult to establish this flow due to the lack of specialized medical experts and to the delayed expert assistance, in addition to the lack of knowledge by police authorities in addressing the issue. 20 Notification to health authorities is also relevant in the preparation of public policies and healthcare, as observed by Gaspar et al. 27 in a research on the notification of sexual violence in Brazil between 2009 and 2013.

Sexual violence was the first sexual experience of a significant number of adolescents, as reported by the victims and observed by the recent hymen rupture on medical examination. This data corroborates the study by Facuri et al., 28 which evaluated victims of sexual violence in a university referral service in the state of São Paulo, Brazil. There is, therefore, great damage to the sexual health of these adolescents, since the literature reports that these women may develop risky sexual behaviors, especially for the acquisition of STIs, drug abuse, and psychiatric disorders. 22 In addition, this population is more likely to suffer repetitive violence, as seen by Delziovo et al., 29 especially for being proportionally more attacked by someone closely related to them.

The SEAVIDAS-HCRP follows the protocol of the Brazilian Ministry of Health for prophylaxis procedures after sexual violence. The time interval between the violent act and the beginning of prophylaxis is essential both to indicate the use and to obtain the necessary effectiveness to reduce damages such as STIs and pregnancy resulting from rape. The present study showed that ∼ 80% of the victims of sexual violence arrived at the service for assistance within the first 72 hours, and more than half arrived within 24 hours after the traumatic event, optimizing the prescription of HIV ART and emergency contraception.

The acceptance to ART by the patients was considered adequate and higher than the one registered in similar studies in Brazil, such as the one by Figueiredo et al., 30 and in the international literature, such as the studies by Linden et al. 31 and Muriuki et al. 32 It is also important to provide clinical and laboratory follow-up during the 28 days of medication use, since 2 patients needed to adapt the ART due to hematological changes.

During the 10 years of the survey conducted in the present study, different ART schemes were recommended by the Ministry of Health aiming at greater efficacy and reduced side effects. This factor makes it difficult to analyze side effects in the victims assessed in the present study, since the ART schemes used were different over the years. According to studies such as the one by Malinverni et al., 33 the drugs used in ART can be a modifying factor for adequate use. However, the complete structuring of the SEAVIDAS-HCRP, based on multidisciplinary care, was fundamental for the high acceptance of the prescribed prophylaxis by victims of violence. This conclusion was also reported in the study by Nisida, 34 who observed an increased adequate use of prophylaxis in victims who were part of the multidisciplinary care offered by the reception service for victims of sexual violence in the city of São Paulo state of São Paulo, Brazil.

The results of the present study highlight that a small percentage of women participated in the outpatient monitoring, half of whom returned in the 1 ^st^ month, and with only a third returning in the 3 ^rd^ postviolence month. Follow-up is encouraged by the SEAVIDAS-HCRP team to monitor possible seroconversion to STIs and to detect women who will develop mental health disorders, such as depression or post-traumatic stress disorder, requiring a differentiated approach. However, after the prophylaxis is completed, many victims of violence do not return, possibly because when returning to the service they remember the trauma suffered.

The women who underwent follow-up had highly effective interventions, since no victim had seroconversion to HIV, to Hepatitis B or C, or syphilis. Only one patient developed pregnancy. This patient had not received emergency contraception, as she arrived at the service after the 5 ^th^ day of the traumatic event. The pregnancy diagnosis occurred at ∼ 20 ^th^ week and she decided not to terminate the pregnancy legally, even though this possibility was offered by the service. These results reinforce the importance of immediate assistance in the health service center, trained to intervene with prophylaxis, as was also observed in the studies by Facuri et al. 28 and Delziovo et al., 29 conducted in large Brazilian cities and with a sample profile similar to the one used in the present survey.

The results of the present study corroborate the importance of the referral service fulfilling its function of welcoming and helping victims of sexual violence, both in the severe episode and in following-up these women in the first months after the traumatic event. The multidisciplinary characteristic of the service, including physicians, psychologists, social workers, and nurses, certainly contributes to the excellent results observed in the present study. 35 Furthermore, the fact that the SEAVIDAS-HCRP is linked to the Hospital das Clínicas da Faculdade de Medicina de Ribeirão Preto da Universidade de São Paulo makes it possible to insert this important topic in training sessions, especially for health professionals, in the context of undergraduate health courses as well as in medical and multiprofessional residency, sensitizing professionals to the importance of the topic and enabling them to provide the best and most effective care. In addition to this, it is essential to disseminate the service, to provide information at the places that help victims of violence, and to facilitate access to these services.

The main limitations of the present study are its retrospective nature, so it depends on data obtained from medical records. Another limitation is the short monitoring of victims of sexual violence. Data collection from medical records is flawed due to incomplete records, and a long-term follow-up could bring more information to the research.

## Conclusion

In the present cohort, the profile of the victims is young, white women with low education. Regarding the traumatic event, it occurs more at night and is committed by an unknown offender. Acceptance to prophylaxis was high and achieved the desired effect with STI prevention, and a case of pregnancy was registered in a victim who did not receive emergency contraception. The results of the present study show that the organization of health services is essential to help victims of sexual violence and to have an established and safe referral flow for these victims.
